# The advent of precision epigenetics for medulloblastoma

**DOI:** 10.18632/oncoscience.507

**Published:** 2020-05-14

**Authors:** Chaoxi Li, Erwin G. Van Meir

**Affiliations:** ^1^Department of Neurosurgery, University of Alabama at Birmingham, Birmingham, Alabama, USA; ^2^O’Neal Comprehensive Cancer Center, University of Alabama at Birmingham, Birmingham, Alabama, USA

**Keywords:** adhesion G protein-coupled receptor, brain angiogenesis Inhibitor, polycomb repressive complex, EZH2, MBD2

Medulloblastoma (MB) is the most common and fatal malignant pediatric brain tumor, is located in the cerebellum and is associated with significant therapy-related morbidity [1]. MB can be subdivided into four clinically and molecularly distinct groups: wingless (WNT), sonic hedgehog (SHH), and groups 3 and 4 [2]. The standard of care for MB patients irrespective of group consists of surgical resection, cranio-spinal radiation and combination chemotherapy. This intensive regimen improves survival, especially in WNT-MB patients. SHH and group 4 patients have intermediate benefit, while most group 3 patients relapse and die from the disease. Moreover, therapy-induced damage to the developing brain remains a significant problem: young survivors suffer from life-long cognitive, neurological and neuroendocrine side effects. Less toxic novel therapies are urgently needed to improve quality of life of these children and young adults.

Genomic studies have identified frequent alterations in epigenetic regulators in MBs, suggesting that incorporating epigenetic reprogramming therapy into the standard of care for MB patients may be beneficial and potentially less toxic. Polycomb group proteins are transcriptional repressors essential for normal gene regulation during development and are perturbed in a wide range of human cancers. They usually belong to either of two protein complexes: Polycomb Repressive Complex 1 (PRC1) that adds a ubiquityl moiety to histone H2A at lysine 119 (H2AK119ubl) and PRC2 that catalyzes the addition of one to three methyl groups to histone H3 at lysine 27, leading to H3K27me1, H3K27me2 and H3K27me3 [3]. EZH2, the catalytic component of PRC2, writes the suppressive chromatin marker H3K27me3 and is overexpressed in many cancers including MB from all four groups. Targeting EZH2 has broad antitumor effects in pre-clinical cancer models, and specific drugs (Tazemetostat, GSK2816126 and CPI-1205) targeting EZH2 are currently in clinical trials for lymphomas and advanced solid cancers [4]. However, a challenge with epigenetic therapy is their potential for broad genome reprogramming and inherent risk for unwanted awakening of silent oncogenes. Addressing this challenge requires a more precise understanding of the epigenetic modification mechanisms governing individual gene(s).

Adhesion G protein-coupled receptors (ADGRs) include 33 human transmembrane proteins with long N termini involved in cell-cell and cell-matrix communication. A sub-family of ADGRs comprises Brain-specific angiogenesis inhibitors 1-3 (BAI1-3), all highly expressed in brain. Recent studies have shown that the *ADGRB1-3* genes which encode BAI1-3 are epigenetically silenced or carry genetic mutations in several cancers, including brain cancers [5]. We previously showed that BAI1 expression is downregulated in human MB samples through epigenetic silencing and can be reactivated by targeting MBD2, a methyl-CpG DNA binding protein [5]. Loss of BAI1 expression promotes cancer development by destabilizing p53, as BAI1 normally sequesters the E3 ubiquitin ligase Mdm2, thereby preventing it from initiating p53 degradation [6]. There was epigenetic specificity to *ADGRB1* regulation as MeCP2, another MBD family protein was not involved.

**Figure 1:**
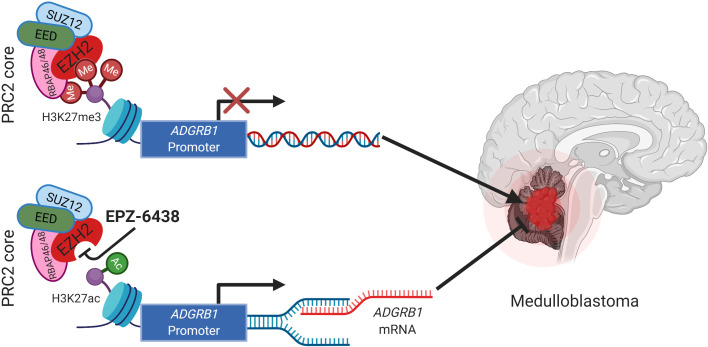
EZH2 inhibitor EPZ-6438 retards medulloblastoma growth through epigenetic reactivation of tumor suppressor gene ADGRB1.

To further investigate the therapeutic impact of targeting histone modifications, we tested EZH2 inhibitor EPZ-6438 (Tazemetostat), and found that it blocked MB cell growth both *in vitro* and *in vivo*, and prolonged survival in orthotopic xenograft models with no obvious toxicity (Figure 1). Remarkably, the therapeutic effect was dependent on epigenetic reactivation of BAI1 expression as shRNA-mediated silencing of *ADGRB1* eliminated the drug benefit. Mechanistically, targeting EZH2 treatment switched the *ADGRB1* gene into an active chromatin status with transition from H3K27me3 to H3K27ac histone marks at the promoter, and recruitment of the C/EBPβ (CREB-binding protein) and CBP transcription factors, which re-activated transcription and BAI1 expression [7].

Interestingly, since we found both MBD2 silencing and EZH2 inhibition could re-activate *ADGRB1* expression, further work is needed to better understand the coordination between DNA and histone modifications in specific epigenetic gene regulation and how to tailor epigenetic targeting for precision treatments. Overall, these new results provide preclinical proof-of-principle for the epigenetic reactivation of BAI1 tumor suppressor in patients with MB.
